# Bt eggplant (*Solanum melongena* L.) in Bangladesh: Fruit production and control of eggplant fruit and shoot borer (*Leucinodes orbonalis* Guenee), effects on non-target arthropods and economic returns

**DOI:** 10.1371/journal.pone.0205713

**Published:** 2018-11-21

**Authors:** M. Z. H. Prodhan, M. T. Hasan, M. M. I. Chowdhury, M. S. Alam, M. L. Rahman, A. K. Azad, M. J. Hossain, Steven E. Naranjo, Anthony M. Shelton

**Affiliations:** 1 Tuber Crops Research Sub Centre, BARI, Bogra, Bangladesh; 2 On Farm Research Division, BARI, Bogra, Bangladesh; 3 BARI, Joydebpur, Gazipur, Bangladesh; 4 Country Coordinator for Bangladesh, USAID Feed the Future South Asia Eggplant Improvement Partnership, Dhaka, Bangladesh; 5 USDA-ARS, Arid-Land Agricultural Research Center, Maricopa, Arizona, United States of America; 6 Department of Entomology, Cornell University/New York State Agricultural Experiment Station (NYSAES), Geneva, New York, United States of America; University of Tennessee, UNITED STATES

## Abstract

Eggplant or brinjal (*Solanum melongena*) is a popular vegetable grown throughout Asia where it is attacked by brinjal fruit and shoot borer (BFSB) (*Leucinodes orbonalis*). Yield losses in Bangladesh have been reported up to 86% and farmers rely primarily on frequent insecticide applications to reduce injury. Bangladesh has developed and released four brinjal varieties producing Cry1Ac (Bt brinjal) and is the first country to do so. We report on the first replicated field trials comparing four Bt brinjal varieties to their non-Bt isolines, with and without standard insecticide spray regimes. Results of the two-year study (2016–17) indicated Bt varieties had increased fruit production and minimal BFSB fruit infestation compared with their respective non-Bt isolines. Fruit infestation for Bt varieties varied from 0–2.27% in 2016, 0% in 2017, and was not significantly affected by the spray regime in either year. In contrast, fruit infestation in non-Bt lines reached 36.70% in 2016 and 45.51% in 2017, even with weekly spraying. An economic analysis revealed that all Bt lines had higher gross returns than their non-Bt isolines. The non-sprayed non-Bt isolines resulted in negative returns in most cases. Maximum fruit yield was obtained from sprayed plots compared to non-sprayed plots, indicating that other insects such as whiteflies, thrips and mites can reduce plant vigor and subsequent fruit weight. Statistically similar densities of non-target arthropods, including beneficial arthropods, were generally observed in both Bt and non-Bt varieties. An additional trial that focused on a single Bt variety and its isoline provided similar results on infestation levels, with and without sprays, and similarly demonstrated higher gross returns and no significant effects on non-target arthropods. Together, these studies indicate that the four Bt brinjal lines are extremely effective at controlling BFSB in Bangladesh without affecting other arthropods, and provide greater economic returns than their non-Bt isolines.

## Introduction

Genetically engineered (GE) crops continue to expand and transform agriculture on a global scale. In 2017, nearly 190M hectares of GE crops were grown by about 18M farmers in 24 countries, including 101M hectares of crops with high levels of host plant resistance to caterpillar and beetle pests [1]. Between 1996 and 2015, this adoption has been associated with increases in farm income > $50,274M and $45,958M, in Bt cotton and maize, respectively, and reductions of > 268M and 87M kg of insecticide active ingredient in Bt cotton and maize, respectively [2]. However, the potential benefits provided by Bt crops have largely gone unrealized in fruits and vegetables where insect management continues to rely primarily on the use of synthetic insecticides [3]. This situation is changing in Bangladesh with the introduction of Bt eggplant.

Eggplant (*Solanum melongena* L.), or brinjal as it is called in Bangladesh and India, is one of the most important and popular vegetables in South and Southeast Asia. The crop is damaged severely by the brinjal fruit and shoot borer (BFSB)(*Leucinodes orbonalis* Guenee) (Lepidoptera: Crambidae). The caterpillar damages brinjal by boring into the petiole and midrib of leaves and tender shoots, resulting in wilting and desiccation of stems. Larvae also feed on flowers, resulting in flower drop or misshapen fruits. The most serious economic damage caused by BFSB is to the fruit, because the holes, feeding tunnels, and larval excrement may make the fruit unmarketable and unfit for human consumption.

BFSB poses a serious problem because of its high reproductive potential, rapid turnover of generations and intensive damage during the wet and dry seasons. Infestation levels may exceed 90% and the yield loss has been estimated up to 86% in Bangladesh [4]. It has been reported that 98% of Bangladeshi farmers rely solely on insecticide sprays to control BFSB [5] and farmers may apply as many as 84 insecticide sprays during the cropping season [6]. This practice is unhealthy for consumers, farmers, and the environment, and is expensive to farmers. As an alternative to intensive use of insecticides, the India-based Maharashtra Hybrid Seed Company (Mahyco) inserted the Cry1Ac gene, under the control of the constitutive 35S CaMV promoter, into eggplant (termed ‘event’ EE-1) to control feeding damage by EFSB. Bt eggplant demonstrated control of EFSB in contained greenhouse trials in India [7]. In late 2003, a partnership was formed between Mahyco, Cornell University, United States Agency for International Development (USAID), and public sector partners in India, Bangladesh, and the Philippines under the Agricultural Biotechnology Support Project II [7]. Mahyco donated the EE-1 event to the Bangladesh Agricultural Research Institute (BARI), where it was incorporated into BARI-developed local varieties. Subsequently, BARI applied to the National Technical Committee on Crop Biotechnology (NTCCB) to release Bt eggplant. Following the recommendation from the NTCCB, the application for release was forwarded to the National Technical Committee on Crop Biotechnology (NTCCB) Core Committee followed by the National Committee on BioSafety (NCB). The Bangladesh government granted approval for release of four varieties on 30 October 2013. On 22 January 2014, Bt seedlings were distributed to 20 farmers in four districts making Bangladesh a pioneer in the world to allow the commercial cultivation of a genetically engineered vegetable crop.

The effective use of this technology requires important knowledge of the agronomic nature of the four varieties and their ability to control BFSB. Furthermore, information is needed on how Bt brinjal affects non-target pest arthropods that likely can affect the yield and quality of brinjal. Performance of the four Bt brinjal varieties and their isolines in spray and no-spray conditions should provide information about the potential damage by these other pests. Likewise, it is important to assess the effect of Bt brinjal on beneficial arthropods, especially biological control agents that can help suppress pest populations. Here we report on the first replicated field trials in Bangladesh to assess the ability of the four Bt brinjal varieties to control BFSB, with and without a standard insecticide regime, compared to their non-Bt isolines. In addition, we assessed plant growth characteristics, economic returns, and potential effects on non-target arthropod pests and on beneficial arthropods that might supply important biological control services.

## Materials and methods

Two sets of complementary experiments were conducted over a two-year period (2016–7) in Bangladesh. In the first experiment, the four commercialized Bt lines were compared to their non-Bt isolines, with and without insecticide sprays to: a) assess their ability to protect the plant from EFSB, b) assess their agronomic characteristics, c) document effects on other arthropods, and d) assess their economic return. In the second experiment, a single Bt line was compared to its isoline, with and without insecticide sprays. This experiment placed more emphasis on assessing the effects of the lines and spray treatments on non-target arthropods, while also assessing the ability of the treatments to control EFSB and provide favorable economic returns.

### Comparisons of four Bt Brinjal varieties and their isolines

#### Plants, sprays and experimental design

Experiments were conducted at the On-Farm Research Division (OFRD) of BARI, in the Bogra District (089^0^22.922^l^ E; 24^0^51.056^l^ N) of Bangladesh. In both years, the experimental field was laid out in a randomized complete block split-plot design with four replications that included insecticide spray regimes as main plots and varieties as sub-plots.

The trials utilized the four Bt brinjal varieties released by BARI to farmers in 2014 [7]: BARI Bt begun-1, BARI Bt begun-2, BARI Bt begun-3, BARI Bt begun-4) and their respective non-Bt isolines (BARI begun-1, BARI begun-2, BARI begun-3, BARI begun-4). In each year, the experimental area was ca. 0.1 ha. Main plots receiving insecticides treatments were 6.0 m x 12.0 m with 3.0 m × 3.0 m sub-plots receiving the brinjal varieties. The distances between sub-plots, main-plots and blocks were 30.0 cm, 1.0 m and 1.5 m, respectively. Row-to-row and plant-to-plant spacing was 100 cm and 75 cm, respectively. Seedlings 35-days old) were transplanted on 12 January 2016 and 10 February 2017.

Sprayed plots were treated weekly with both Admire 20SL (imidacloprid) at 0.5 ml/L of water (active ingredient 50 ml/ha) for sucking arthropods (whiteflies, mites, jassids and aphids) and Proclaim 5SG (emamectin benzoate) at 1 g/L of water (active ingredient 25 g/ha) for BFSB. These two insecticides are commonly used in brinjal production in Bangladesh and weekly, or more frequent, spray schedules are the norm. Sprays were applied using a Knapsack sprayer. Spraying started from crop establishment and continued at weekly intervals to the last harvest, 30 May in 2016 and 25 June in 2017. Before spraying, the non-sprayed plots were covered with a non-porous cloth to prevent spray drift. Non-sprayed plots were sprayed with water only. Fertilizers were used at 138-40-100-18-1.7–3.6 kg/ha (NPKSBZn) and cowdung at10 t/ha. Irrigation, weeding, pruning of side shoots and other cultural operations were done when necessary following standard practices for brinjal production in Bangladesh [8].

#### Measurements and data analysis

Data were collected weekly on plant growth patterns (plant height and width (or bushiness)), number of flowers per plant, percent damaged shoots and fruits by BFSB, and marketable and non-marketable fruits on each of four plants per plot. Arthropod populations (pests and beneficials) were sampled weekly on the five newest leaves on each of four randomly selected plants per plot. The upper and lower surfaces of the leaves were thoroughly examined for the presence of arthropods. All weekly counts were taken from 1 February to 30 May, 2016 and from 1 April to 25 June, 2017. Arthropod counts were made before 9 am. A mixed-model, split-plot ANOVA was used for analyses with block as the random effect and plant type and insecticide as fixed effects. Each year was analyzed separately. The response variable was the seasonal mean for each variable examined over time. Arcsine square-root transformations were applied to percentage data but untransformed means are presented. Mean differences were contrasted using Tukey’s HSD test and analyses were done using the statistical package ‘R’. The total seasonal pesticide load (each insecticide applied × number of applications × dose) was used to estimate the Environmental Impact Quotient (EIQ) [9].

At harvest, fruits were judged as infested or not and given a value according to the current market prices. Non-infested Bt begun-4 and its non-infested isoline was valued at $0.36/kg and the others at $0.30/kg; all infested fruit were valued at $0.02/kg. The variable cost of production was calculated based on the cost of seeds, fertilizers, insecticides and irrigation and was $2,258 for sprayed plots and $1,139 for non-sprayed plots. A partial budgeting analysis was used to estimate gross return and gross margin of profits for each treatment as:

Grossreturn=PQ

Grossmargin=PQ−VC

where, Q is brinjal yield (kg/ha), P is brinjal price ($/kg) and VC are the variable costs ($/ha) associated with crop production. Bangladesh farmers typically are interested in the gross margin and so this metric is reported here.

### Comparisons of one Bt Brinjal variety and its isoline

#### Plants, sprays and experimental design

The experiment was conducted at OFRD, BARI, Bogra (089^0^22.858^′^E; 24^0^51.088^′^N), Bangladesh. In both years, the experiment was laid out in a ca. 0.1 ha field using a randomized complete block design with four replications. Plot size was 4.5m × 9.0 m and the distances between plots and blocks were 30.0 cm and 1.5 m, respectively. Treatments consisted of two varieties of brinjal, BARI Btbegun-1 and BARI begun-1 (non-Bt isoline), each sprayed or unsprayed with insecticides for a total of four treatments. Seedlings were transplanted on January 13, 2016 and on 11 February 2017. Plant spacing, fertilizer use, cultural practices and insecticide sprays were as described above.

#### Measurements and data analysis

Data were taken weekly on percent damaged shoots and fruits by BFSB, marketable and non-marketable fruits, economic returns and densities of arthropods (pest and beneficial arthropods). Arthropod populations were assessed using three methods: 1) Counts on plants were taken from 10 randomly selected plants from the interior of the plot. For each plant, all arthropods were counted on the upper and lower surface of the top 5 leaves and counts were made before 9 am; 2) yellow sticky traps (45×18 cm) were used to measure aerial populations of insects with three traps placed in each plot at crop canopy level; 3) pitfall traps were used to measure ground dwelling arthropods. Three plastic cups (10 cm diameter and 8 cm deep) were placed in the soil in each of the plots with the mouth of the cup at ground level. Each cup was half-filled with water and a few drops of detergent as a trapping fluid. Each week the trapped arthropods were placed into plastic bottles filled with 70% alcohol. Samples were labeled and stored until identified. All counts were done weekly from 2 February to 31 May, 2016 and from 1 April to 25 June, 2017. A mixed-model ANOVA was used with block as the random effect and plant type and insecticides as fixed effects. Each year was analyzed separately. The response variable was the seasonal mean for each variable examined over time. Arcsine square-root transformations were applied to percentage data but untransformed means are presented. Mean differences were contrasted using Tukey’s HSD test and analysis were done using the statistical package ‘R’. The total seasonal pesticide load (each insecticide applied × number of applications × dose) was used to estimate the Environmental Impact Quotient (EIQ) [9]. As before, gross returns and gross margins were estimating with a partial budgeting analysis.

## Results

### Comparisons of four Bt Brinjal varieties and their isolines

#### Infestation by BFSB

In both years, significant differences were observed among the varieties for BFSB infestation (Table 1). Regardless of spray regime, there was no shoot infestation by EFSB in any of the four Bt brinjal varieties in either year, but shoot infestation occurred in all non-Bt brinjal varieties regardless of spray regime. Infested fruit for Bt varieties varied from 0 to 2.27% and was not significantly affected by the spray regime in either year. In contrast, the percent infested fruit in the non-Bt brinjal varieties reached 36.70% in 2016 and 45.51% in 2017 (Table 1). Another measure of fruit infestation was the percent fruit infested by weight, which was more reflective of income because brinjal is sold by weight and infested fruit bring a lower value. By weight, the highest percent of infested Bt brinjal fruit was only 2.27% (2016, BARI Bt begun 2), compared to the highest rate for non-Bt brinjal of 44.30% (2017, non-Bt isoline 1). In some cases, spraying significantly reduced infestation in non-Bt varieties [e.g. 2016, sprayed non-Bt isoline 4 reduced damage by 25.38% (34.49–9.11%) while in other cases spraying did not (e.g. 2016, non-Bt isoline 2 (21.89–19.17%)].

**Table 1:** Mean infestation^1^ in brinjal by brinjal shoot and fruit borer (BFSB) relative to insecticides regimes and variety, OFRD, BARI, Bogra, Bangladesh.

Year	Spray schedule	Variety	Infested shoot(%)	Infested fruit by No. (%)	Infested fruit by wt. (%)
2016	Spray	BARI Bt begun-1	0.00 (0.00) f	0.00 (0.00) d	0.00 (0.00) d
		BARI Bt begun-2	0.00 (0.00) f	2.27 (0.417) cd	2.27 (0.416) cd
		BARI Bt begun-3	0.00 (0.00) f	1.89 (0.312) cd	0.91 (0.263) cd
		BARI Bt begun-4	0.00 (0.00) f	0.38 (0.17) cd	1.06 (0.284) cd
		Non-Bt isoline-1	0.82 (0.073) de	35.87 (0.128) a	33.72 (0.131) a
		Non-Bt isoline-2	0.35 (0.179) ef	27.78 (0.204) a	21.89 (0.199) ab
		Non-Bt isoline-3	2.76 (0.047) abc	24.92 (0.105) ab	22.93 (0.122) ab
		Non-Bt isoline-4	2.41 (0.136) bc	36.70 (0.53) a	34.49 (0.492) a
	No-spray	BARI Bt begun-1	0.00 (0.00) f	2.27 (0.417) cd	0.24 (0.136) d
		BARI Bt begun-2	0.00 (0.00) f	0.00 (0.00) d	0.00 (0.00) d
		BARI Bt begun-3	0.00 (0.00) f	1.14 (0.295) cd	1.14 (0.294) cd
		BARI Bt begun-4	0.00 (0.00) f	0.00 (0.00) d	0.00 (0.00) d
		Non-Bt isoline-1	1.57 (0.189) cd	21.97 (0.262) ab	20.95 (0.225) ab
		Non-Bt isoline-2	0.74 (0.169) de	22.14 (0.129) ab	19.17 (0.150) ab
		Non-Bt isoline-3	4.49 (0.216) a	18.79 (0.188) ab	16.90 (0.226) ab
		Non-Bt isoline-4	3.34 (0.147) ab	9.09 (0.501) bc	9.11 (0.502) bc
2017	Spray	BARI Bt begun-1	0.00 (0.00) c	0.00 (0.00) d	0.00 (0.00) d
		BARI Bt begun-2	0.00 (0.00) c	0.00 (0.00) d	0.00 (0.00) d
		BARI Bt begun-3	0.00 (0.00) c	0.00 (0.00) d	0.00 (0.00) d
		BARI Bt begun-4	0.00 (0.00) c	0.00 (0.00) d	0.00 (0.00) d
		Non-Bt isoline-1	1.14 (0.32) b	45.51 (0.03) a	44.30 (0.02) a
		Non-Bt isoline-2	0.61 (0.06) b	34.25 (0.08) ab	36.88 (0.08) ab
		Non-Bt isoline-3	2.24 (0.09) a	41.79 (0.25) a	40.14 (0.21) a
		Non-Bt isoline-4	1.60 (0.12) ab	29.82 (0.33) ab	32.38 (0.45) ab
	No-spray	BARI Bt begun-1	0.00 (0.00) c	0.00 (0.00) d	0.00 (0.00) d
		BARI Bt begun-2	0.00 (0.00) c	0.00 (0.00) d	0.00 (0.00) d
		BARI Bt begun-3	0.00 (0.00) c	0.00 (0.00) d	0.00 (0.00) d
		BARI Bt begun-4	0.00 (0.00) c	0.00 (0.00) d	0.00 (0.00) d
		Non-Bt isoline-1	1.43 (0.14) ab	36.97 (0.34) a	35.21 (0.41) ab
		Non-Bt isoline-2	0.92 (0.13) ab	18.38 (0.43) bc	18.31 (0.38) bc
		Non-Bt isoline-3	1.75 (0.16) ab	11.33 (0.35) c	10.93 (0.37) c
		Non-Bt isoline-4	1.39 (0.10) ab	11.47 (0.28) c	10.99 (0.23) c

^1^ Figures in parenthesis are SE values; means followed by the same letter in a column within a year do not differ significantly by HSD at 5% level

#### Yields and gross margins

In both years, significant differences were evident in the economic returns due to BFSB infestation and costs for spraying (Table 2). In both years all Bt lines has higher gross margins than their isolines, regardless of whether they were sprayed or not. In 2016, all four Bt brinjal varieties showed a positive gross margin, even when no sprays were applied. In contrast only two of the non-Bt isolines that were sprayed showed a positive gross margin when sprayed and only one of the unsprayed non-Bt isolines showed a positive gross margin. In 2017, all of the non-sprayed, non- Bt isolines had negative gross margins, as did one of the non-sprayed Bt brinjal varieties (BARI Bt begun-4). In 2016, spraying Bt brinjal varieties always improved the gross margin, despite the higher production costs. Spraying a non-Bt isoline could also improve its gross margin, but not to the level of the sprayed Bt variety (e.g., in 2016 the gross margin for sprayed Bt begun-2 was $7,634.74 compared to its sprayed non-Bt isoline of $2,458.00).

**Table 2:** Mean yields^1^ and economic returns for brinjal by brinjal shoot and fruit borer (BFSB) relative to insecticides and variety, OFRD, BARI, Bogra, Bangladesh.

Year	Spray schedule	Variety	Fruit yield(t/ha)		Gross return ($)/ha^2^	Gross margin ($)/ha^2^
			Non-infested	Infested		
2016	Spray	BARI Bt begun-1	16.76 (2.56) bcd	0.00 (0.00) c	4,984.22 (763.10) bcd	2,726.04 (763.10) bcd
		BARI Bt begun-2	33.23 (2.89) a	0.33 (0.33) bc	9,892.92 (855.13) a	7,634.74 (855.13) a
		BARI Bt begun-3	21.22 (2.45) b	0.08 (0.08) c	6,312.30 (727.92) b	4,054.12 (727.92) b
		BARI Bt begun-4	15.49 (1.09) bcd	0.38 (0.30) bc	5,537.39 (387.74) bc	3,279.21 (387.74) bcd
		Non-Bt isoline-1	6.43 (0.39) efg	3.02 (0.53) abc	1,984.97 (125.56) ef	-273.21 (125.56) e
		Non-Bt isoline-2	15.55 (0.92) bcd	3.81 (0.38) ab	4,716.18 (269.72) bcd	2,458.00 (269.72) bcd
		Non-Bt isoline-3	11.92 (0.88) cde	4.36 (0.48) a	3,648.74 (273.36) cde	1,390.56 (273.36) cde
		Non-Bt isoline-4	5.49 (1.11) efg	3.41 (0.83) abc	2,040.93 (397.17) ef	-217.24 (397.17) e
	No-spray	BARI Bt begun-1	9.39 (1.10) def	0.05 (0.05) c	2,794.25 (327.96) def	1,655.05 (327.96) cde
		BARI Bt begun-2	19.08 (2.98) bc	0.00 (0.00) c	5,673.71 (887.79) bc	4,534.50 (887.79) b
		BARI Bt begun-3	16.06 (2.46) bcd	0.04 (0.04) c	4,777.41 (732.76) bcd	3,638.21 (732.76) b
		BARI Bt begun-4	6.46 (1.02) efg	0.05 (0.04) c	2,305.35 (367.12) ef	1,166.15 (367.12) de
		Non-Bt isoline-1	3.45 (1.33) fg	2.07 (0.79) abc	1,075.60 (410.48) f	-63.60 (410.48) e
		Non-Bt isoline-2	6.86 (1.86) efg	2.11 (0.49) abc	2,090.59 (557.80) ef	951.39 (557.80) de
		Non-Bt isoline-3	3.65 (1.43) fg	1.87 (0.99) abc	1,129.39 (449.40) f	-9.82 (449.40) e
		Non-Bt isoline-4	1.36 (0.84) g	3.11 (2.23) abc	560.91 (354.57) f	-578.29 (354.57) e
2017	Spray	BARI Bt begun-1	15.44 (3.59) c	0.24 (0.17) e	4,599.27 (1068.48) bc	2,341.091068.48bcd
		BARI Bt begun-2	36.61 (4.28) a	0.15 (0.04) e	1,0894.07 (1274.29) a	8,635.89 (1274.29) a
		BARI Bt begun-3	22.11 (4.99) b	0.11 (0.04) e	6,580.77 (1484.04) b	43,228.00 (1484.04) b
		BARI Bt begun-4	16.74 (3.15) c	0.07 (0.02) e	5,976.10 (1125.80) b	3717.92 (1125.80) bc
		Non-Bt isoline-1	5.34 (0.37) ef	8.40 (1.33) a	1,789.91 (102.34) cde	-468.27 (102.34) de
		Non-Bt isoline-2	14.14 (2.28) c	6.82 (1.07) b	4,368.46 (682.30) bcd	2,110.28 (682.30) bcd
		Non-Bt isoline-3	9.03 (1.35) d	8.23 (1.02) a	2,882.58 (404.23) cde	624.40 (404.23) de
		Non-Bt isoline-4	3.41 (0.53) fg	5.66 (1.13) b	1,350.72 (198.78) e	-907.46 (198.78) e
	No-spray	BARI Bt begun-1	5.42 (0.68) ef	0.04 (0.02) e	1,612.86 (202.21) de	473.65 (202.21) de
		BARI Bt begun-2	6.70 (1.29) de	0.06 (0.004) e	1,993.83 (383.94) cde	854.63 (383.94) cde
		BARI Bt begun-3	8.59 (1.77) d	0.01 (0.008) e	2,555.92 (526.71) cde	1,416.72 (526.71) bcde
		BARI Bt begun-4	2.39 (0.61) fg	0.01 (0.008) e	853.77 (218.52) cde	-285.44 (218.52) de
		Non-Bt isoline-1	3.58 (2.44) fg	1.97 (0.37) cd	1,112.27 (726.41) e	-26.93 (726.41) de
		Non-Bt isoline-2	2.06 (0.73) g	0.96 (0.59) de	635.99 (226.43) e	-503.22 (226.43) de
		Non-Bt isoline-3	2.49 (0.77) fg	1.83 (0.85) cd	785.53 (240.21) e	-353.67 (240.21) de
		Non-Bt isoline-4	1.71 (0.68) g	2.92 (1.59) c	679.12 (211.89) e	-460.08 (211.89) de

^1^ Figures in parenthesis are SE values; means followed by the same letter in a column within a year do not differ significantly by HSD at 5% level

^2^ Market price of brinjal: Non-infested BARI Bt begun-4 @ $0.36/kg, others @ $0.30/kg and infested @ $0.02/kg. Cost of spray: two laborers/spray/ha @ Tk. $3.56/labor; Urea@ $0.19/kg, TSP@ $0.26/kg, MP@ $0.18/kg, Zypsum @ $0.07/kg, Boric acid @ $1.78/kg, Zn @ @3.96/kg, Cowdung @ $0.01/kg. Admire @ $17.8/250 ml and Proclaim @ 26.77/500 ml. 1 US dollar = 84.05 Bangladeshi Taka (29 March 2018)

#### Effects on non-target pest arthropods

Eleven different non-target pest arthropods were observed. Five sucking pests, including whitefly (*Bemisia tabaci* Gennadius), thrips (*Thrips palmi* Karny), aphid (*Aphis gossypii* Glover), jassid (*Amrasca biguttula biguttula* Ishida) and mites (*Tetranychus urticae* Koch) had populations > 0.2 per leaf per and were analyzed (Table 3). Populations of flea beetle (*Phyllotreta striolata*), armyworms (*Spodoptera litura*), Mirid bug (*Helopeltis* sp.), Epilachna beetle (*Epilachna* sp.) and stink bug (*Nezara* sp.) were too low (< 0.01) for meaningful analysis. In 2016, spraying generally significantly increased populations of whiteflies and mites but decreased populations of thrips, aphids and jassids. While there were significant differences in other non-target pest populations, they were always at low densities and effects were likely of little consequence. In 2017, generally there were statistically higher populations of whiteflies, but statistically lower populations of aphids and jassids in sprayed plots. Mites were not present in 2017 probably because of the wetter weather. Variety did not have a consistent effect on densities of non-target pest arthropods.

**Table 3:** Mean abundance^1^ of non-target pest arthropods (No./leaf) in brinjal relative to insecticides and varieties, OFRD, BARI, Bogra, Bangladesh.

Year	Spray schedule	Variety	Whitefly^2^	Thrips^3^	Aphid^4^	Jassid^5^	Mite^6^
2016	Spray	BARI Bt begun-1	3.25 (0.29) bc	1.15 (0.04) fg	0.54 (0.02) c	0.27 (0.02) c	6.48 (0.64) ab
		BARI Bt begun-2	2.37 (0.24) c	1.22 (0.11) fg	0.55 (0.04) c	0.21 (0.007) c	5.84 (0.65) abc
		BARI Bt begun-3	3.18 (0.26) bc	1.28 (0.09) efg	0.46 (0.07) c	0.28 (0.05) c	7.05 (0.76) ab
		BARI Bt begun-4	4.03 (0.37) b	0.97 (0.04) g	0.58 (0.03) c	0.20 (0.01) c	6.0 (0.88) abc
		Non-Bt isoline-1	3.39 (0.45) b	1.20 (0.04) fg	0.51 (0.004) c	0.27 (0.02) c	6.51 (0.86) ab
		Non-Bt isoline-2	3.21 (0.59) bc	1.53 (0.11) cdef	0.56 (0.05) c	0.26 (0.04) c	6.23 (0.98) ab
		Non-Bt isoline-3	4.05 (0.21) b	1.45 (0.09) def	0.63 (0.10) bc	0.24 (0.02) c	8.23 (1.09) a
		Non-Bt isoline-4	5.14 (0.48) a	1.14 (0.03) fg	0.69 (0.05) bc	0.27 (0.03) c	6.25 (0.67) ab
	No-spray	BARI Bt begun-1	0.95 (0.08) d	1.81 (0.10) abcd	1.74 (0.26) a	0.69 (0.06) b	3.49 (0.48) de
		BARI Bt begun-2	0.93 (0.07) d	1.93 (0.20) abc	1.74 (0.14) a	0.96 (0.09) ab	3.39 (0.41) de
		BARI Bt begun-3	1.08 (0.25) d	2.03 (0.05) ab	1.29 (0.09) ab	0.81 (0.06) ab	4.94 (1.09) bcde
		BARI Bt begun-4	1.31 (0.14) d	1.74 (0.16) abcde	1.57 (0.13) a	0.95 (0.19) ab	3.42 (0.19) de
		Non-Bt isoline-1	1.16 (0.13) d	1.69 (0.07) bcde	1.69 (0.21) a	1.01 (0.12) ab	3.59 (0.24) cde
		Non-Bt isoline-2	1.13 (0.06) d	1.84 (0.05) abcd	1.74 (0.09) a	1.14 (0.06) a	3.24 (0.38) e
		Non-Bt isoline-3	1.34 (0.13) d	2.15 (0.08) a	1.61 (0.23) a	0.78 (0.12) b	4.97 (0.81) bcde
		Non-B tisoline-4	1.41 (0.15) d	1.82 (0.09) abcd	1.64 (0.12) a	0.89 (0.05) ab	4.75 (0.46) bcde
2017	Spray	BARI Bt begun-1	0.72 (0.03) cd	1.34 (0.16) ab	0.29 (0.07) cd	0.26 (0.02) c	NA^7^
		BARI Bt begun-2	0.72 (0.03) cd	1.92 (0.05) a	0.31 (0.03) bcd	0.34 (0.03) c	NA
		BARI Bt begun-3	0.79 (0.07) bc	1.37 (0.16) ab	0.22 (0.04) d	0.34 (0.04) c	NA
		BARI Bt begun-4	0.99 (0.08) ab	1.32 (0.17) ab	0.30 (0.03) bcd	0.30 (0.02) c	NA
		Non-Bt isoline-1	0.99 (0.09) ab	1.36 (0.11) ab	0.30 (0.05) bcd	0.35 (0.04) c	NA
		Non-Bt isoline-2	0.63 (0.05) cde	1.22 (0.09) ab	0.30 (0.01) bcd	0.37 (0.03) c	NA
		Non-Bt isoline-3	0.81 (0.08) bc	1.40 (0.12) ab	0.31 (0.04) bcd	0.34 (0.04) c	NA
		Non-Bt isoline-4	1.06 (0.11) a	1.140 (0.08) b	0.33 (0.02) bcd	0.33 (0.01) c	NA
	No- spray	BARI Bt begun-1	0.36 (0.04) f	1.55 (0.07) ab	0.46 (0.07) abcd	0.84 (0.04) ab	NA
		BARI Bt begun-2	0.36 (0.03) f	1.54 (0.28) ab	0.69 (0.15) a	0.73 (0.07) b	NA
		BARI Bt begun-3	0.39 (0.04) f	1.24 (0.07) ab	0.65 (0.10) a	0.64 (0.05) b	NA
		BARI Bt begun-4	0.51 (0.03) def	1.63 (0.18) ab	0.57 (0.03) ab	0.75 (0.02) b	NA
		Non-Bt isoline-1	0.46 (0.015) ef	1.25 (0.07) ab	0.61 (0.13) a	0.82 (0.05) ab	NA
		Non-Bt isoline-2	0.41 (0.02) ef	1.12 (0.03) b	0.53 (0.09) abc	0.64 (0.03) b	NA
		Non-Bt isoline-3	0.45 (0.02) ef	1.55 (0.21) ab	0.51 (0.08) abc	0.65 (0.04) b	NA
		Non-Bt isoline-4	0.44 (0.02 ef	1.44 (0.13) ab	0.63 (0.08) a	1.00 (0.10) a	NA

^1^Figures in parenthesis are SE values; means followed by the same letter in a column within a year do not differ significantly by HSD at 5% level

^2^*Bemisia tabaci* Gennadius,

^3^*Thrips palmi* Karny,

^4^*Aphis gossypii* Glover,

^5^*Amrasca biguttula biguttula* Ishida,

^6^*Tetranychus urticae* Koch

^7^NA, data not taken because of extremely low populations.

#### Effects on non-target beneficial arthropods

Ladybird beetles and spiders were the most abundant beneficial arthropods but still only reached 0.067 beetles/leaf and 0.030 spiders/leaf in 2016 (Table 4). Populations of red ant (*Solenopsis* sp.), rove beetle (*Homaeotarsus* sp.), assassin bug (*Zelus* sp.), ground beetle (*Ophionia nigrofasciata*,), syrphid fly (*Syrphus* sp.) and small black ant (*Camponotus* sp.) were too low (< 0.01) for meaningful analysis. In 2016 spraying sometimes, but not consistently, significantly reduced the populations of *Coccinella* sp. but not spiders. In 2017, there were no significant differences between populations of *Coccinella* and spiders on a variety in sprayed and non-sprayed plots. Variety did not have a consistent effect on non-target beneficial arthropods.

**Table 4:** Mean abundance^1^ of non-target beneficial arthropods (No./leaf) in brinjal relative to insecticides and varieties, OFRD, BARI, Bogra, Bangladesh.

Year	Spray schedule	Variety	Ladybird beetles^2^	Spiders^3^
2016	Spray	BARI Bt begun-1	0.021 (0.005) bcde	0.017 (0.006) a
		BARI Bt begun-2	0.007 (0.003) e	0.018 (0.003) a
		BARI Bt begun-3	0.011 (0.003) de	0.020 (0.006) a
		BARI Bt begun-4	0.017 (0.004) bcde	0.027 (0.002) a
		Non-Bt isoline-1	0.013 (0.002) cde	0.015 (0.005) a
		Non-Bt isoline-2	0.015 (0.002) cde	0.015 (0.002) a
		Non-Bt isoline-3	0.024 (0.003) bcde	0.018 (0.001) a
		Non-Bt isoline-4	0.015 (0.004) cde	0.020 (0.006) a
	No- spray	BARI Bt begun-1	0.034 (0.014) bcde	0.030 (0.008) a
		BARI Bt begun-2	0.032 (0.006) bcde	0.015 (0.002) a
		BARI Bt begun-3	0.067 (0.01) a	0.020 (0.003) a
		BARI Bt begun-4	0.031 (0.005) bcde	0.015 (0.002) a
		Non-Bt isoline-1	0.040 (0.005) abcd	0.021 (0.003) a
		Non-Bt isoline-2	0.042 (0.004) abc	0.013 (0.002) a
		Non-Bt isoline-3	0.037 (0.002) abcd	0.026 (0.003) a
		Non-Bt isoline-4	0.046 (0.007) ab	0.028 (0.002) a
2017	Spray	BARI Bt begun-1	0.007 (0.002) a	0.039 (0.007) abc
		BARI Bt begun-2	0.005 (0.003) a	0.025 (0.007) c
		BARI Bt begun-3	0.006 (0.002) a	0.032 (0.006) abc
		BARI Bt begun-4	0.012 (0.004) a	0.027 (0.005) bc
		Non-Bt isoline-1	0.007 (0.002) a	0.029 (0.005) abc
		Non-Bt isoline-2	0.006 (0.003) a	0.023 (0.003) c
		Non-Bt isoline-3	0.004 (0.002) a	0.022 (0.003) c
		Non-Bt isoline-4	0.006 (0.002) a	0.034 (0.004) abc
	No- spray	BARI Bt begun-1	0.013 (0.002) a	0.051 (0.004) ab
		BARI Bt begun-2	0.013 (0.003) a	0.044 (0.006) abc
		BARI Bt begun-3	0.015 (0.006) a	0.043 (0.005) abc
		BARI Bt begun-4	0.012 (0.002) a	0.045 (0.004) abc
		Non-Bt isoline-1	0.015 (0.004) a	0.0520.006) a
		Non-Bt isoline-2	0.007 (0.001) a	0.032 (0.001) abc
		Non-Bt isoline-3	0.012 (0.003) a	0.045 (0.007) abc
		Non-Bt isoline-4	0.014 (0.003) a	0.042 (0.005) abc

^1^ Figures in parenthesis are SE values; means followed by the same letter in a column within a year do not differ significantly by HSD at 5% level

^2^ *Coccinella* sp.,

^3^*Oxyopes* sp. plus *Tetragnatha* sp.

#### Variety characteristics

There were some significant differences in plant height, bushiness (width) and number of shoots and flowers per plant between Bt varieties and their non-Bt isolines (Table 5). However, there were no clear trends that Bt plants differed from their isolines in height or bushiness, although it has been suggested that BFSB-infested shoots may affect plant architecture by killing stems. Likewise, spraying did not appear to have a consistent effect on plant characteristics. The most dramatic and consistent differences were the number of fruit per plant. Generally, Bt plants had significantly more fruit per plant than their respective non-Bt isolines in both years.

**Table 5:** Mean values^1^ of brinjal agronomic characteristics relative to insecticides and varieties, OFRD, BARI, Bogra, Bangladesh.

Year	Spray schedule	Variety	Plant height (cm)	Plant width Bushiness (cm)	Shoots/plant (No.)	Flowers/plant (No.)	Fruits/plant (No.)
2016	Spray	BARI Bt begun-1	44.28 (2.32) cde	73.79 (1.33) abcd	9.08 (0.57) efg	89.44 (3.65) abc	72.0 (4.85) b
		BARI Bt begun-2	53.45 (0.20) ab	90.58 (1.54) a	14.22 (0.18) a	110.69 (5.01) a	114.15 (12.74) a
		BARI Bt begun-3	47.03 (1.14) bcd	81.77 (3.48) abc	10.30 (0.84) cdef	84.31 (7.12) abcd	33.13 (2.43) cd
		BARI Bt begun-4	49.90 (0.44) abc	84.19 (1.71) abc	14.07 (1.59) ab	37.88 (2.35) ef	12.88 (1.43) de
		Non-Bt isoline-1	42.32 (0.88) def	83.91 (1.49) abc	12.68 (0.72) abc	111.81 (4.81) a	24.44 (2.73) cde
		Non-Bt isoline-2	44.03 (1.90) cde	88.77 (2.19) ab	14.62 (0.29) a	117.31 (3.87) a	42.35 (2.27) c
		Non-Bt isoline-3	54.27 (1.25) a	85.11 (2.11) abc	12.13 (0.39) abcd	67.06 (6.07) bcde	16.02 (1.74) de
		Non-Bt isoline-4	56.37 (1.42) a	90.41 (2.56) a	12.78 (0.52) abc	39.10 (2.34) ef	5.25 (1.17) e
	No-spray	BARI Bt begun-1	35.66 (1.29) fg	61.30 (3.56) d	7.20 (0.64) g	52.31 (6.29) cdef	29.02 (4.17) cd
		BARI Bt begun-2	43.41 (1.62) cde	87.81 (11.53) ab	12.80 (0.63) abc	98.56 (29.63) ab	77.19 (3.10) b
		BARI Bt begun-3	39.21 (1.76) efg	67.70 (1.64) cd	9.02 (0.57) fg	48.88 (5.36) cdef	17.44 (3.70) de
		BARI Bt begun-4	42.38 (0.73) def	72.41 (2.01) abcd	10.69 (0.34) cdef	18.75 (1.45) f	5.38 (1.45) e
		Non-Bt isoline-1	33.54 (1.40) g	71.44 (4.08) bcd	9.73 (0.90) defg	55.13 (5.60) cdef	20.06 (3.92) cde
		Non-Bt isoline-2	33.01 (1.18) g	72.69 (2.34) abcd	11.93 (0.64) abcde	55.90 (5.59) bcdef	23.19 (7.07) cde
		Non-Bt isoline-3	45.92 (2.06) cde	76.80 (2.79) abcd	11.23 (0.81) bcdef	42.94 (3.98) def	5.44 (1.29) e
		Non-Bt isoline-4	44.38 (1.88) cde	77.02 (3.39) abcd	12.41 (1.09) abcd	22.44 (2.45) f	2.06 (0.89) e
2017	Spray	BARI Bt begun-1	59.36 (0.59) cdef	69.35 (2.41) efgh	12.96 (0.37) de	123.59 (10.64) abc	51.33 (7.09) b
		BARI Bt begun-2	67.57 (1.34) ab	81.75 (2.31) abc	17.22 (0.72) ab	114.50 (2.95) abcd	106.42 (5.83) a
		BARI Bt begun-3	55.93 (1.85) efgh	71.62 (2.57) cdefg	13.75 (0.65) cd	98.16 (9.33) bcde	23.56 (2.33) cd
		BARI Bt begun-4	71.74 (1.67) a	82.12 (1.22) ab	15.71 (0.54) abc	43.69 (2.26) fgh	17.06 (6.96) cdefg
		Non-Bt isoline-1	57.64 (2.21) defg	82.16 (1.68) ab	17.22 (0.50) ab	151.47 (10.08) a	19.56 (2.76) cdef
		Non-Bt isoline-2	63.84 (2.57) bcd	86.08 (2.13) a	18.25 (0.94) a	134.31 (23.77) ab	27.47 (2.33) c
		Non-Bt isoline-3	66.32 (3.36) abc	79.71 (2.75) abcd	14.55 (0.63) cd	87.09 (6.40) cde	8.81 (2.58) defg
		Non-Bt isoline-4	73.32 (1.57) a	84.96 (1.46) a	14.97 (0.64) bcd	36.03 (3.80) gh	4.71 (1.30) efg
	No-spray	BARI Bt begun-1	50.73 (1.54) ghi	62.54 (2.04) gh	10.86 (0.60) ef	67.91 (3.88) efg	20.25 (3.46) cde
		BARI Bt begun-2	52.84 (1.69) fgh	66.62 (2.01) fgh	12.81 (0.86) de	61.09 (3.75) efg	18.65 (3.36) cdefg
		BARI Bt begun-3	45.052.15) i	60.44 (6.37) h	9.89 (0.92) f	48.16 (2.86) fgh	12.15 (1.46) cdefg
		BARI Bt begun-4	63.35 (2.12) bcde	73.58 (3.05) bcdef	14.22 (0.91) cd	20.81 (2.50) h	2.57 (0.70) fg
		Non-Bt isoline-1	51.97 (0.19) fghi	77.90 (1.02) abcde	14.75 (0.66) bcd	78.62 (9.46) def	3.34 (0.91) efg
		Non-Bt isoline-2	48.58 (1.07) hi	71.73 (2.48) cdefg	15.78 (0.61) abc	66.81 (7.61) efg	4.75 (1.87) efg
		Non-Bt isoline-3	52.64 (1.21) fghi	69.18 (1.78) efgh	12.96 (0.78) de	43.46 (3.27) fgh	4.31 (1.70) efg
		Non-Bt isoline-4	55.05 (3.48) fgh	71.12 (2.94) defg	12.73 (0.75) de	21.16 (2.43) h	2.26 (0.74) g

^1^ Figures in parenthesis are SE values; means followed by the same letter in a column within a year do not differ significantly by HSD at 5% level

#### Environmental impact quotient

In both years, the same number of sprays (19) was applied to sprayed plots. The seasonal insecticide load/ha for imidacloprid and emamectin benzoate was 36.7 and 26.3 mg active ingredient per ha, respectively, and the seasonal calculated EIQ values for imidacloprid were 8.4 (consumer), 5.6 (field worker) and 75.5 (ecological) and for emamectin benzoate 1.7 (consumer), 3.8 (farmer worker) and 27.9 (ecological).

### Comparisons of one Bt Brinjal varieties and its isoline

#### Infestation by BFSB

In both years, in all but one case (i.e., sprayed plots in 2016) there were significant differences in infested shoots and fruit for Bt begun-1 compared to its isoline (Table 6). In 2016, percent infested fruit for Bt begun-1varied between 0–0.16% depending on whether it was sprayed or not, while its non-Bt isoline had infestation rates between 39.33 and 50.85% when sprayed or not, respectively. In 2017, similar lack of infestation of Bt begun-1 fruit was observed whether it was sprayed or not, while its isoline had 41.52% infested fruit when sprayed and 52.43% when not sprayed (Table 6).

**Table 6:** Mean infestation^1^ in brinjal by brinjal shoot and fruit borer (BFSB) relative to insecticides and varieties, OFRD, BARI, Bogra, Bangladesh.

Year	Spray schedule	Variety	Infested shoot (%)	Infested fruit (by No.) (%)	Infested fruit (by wt) (%)
2016	Spray	BARI Bt begun-1	0.00 (0.00) b	0.16 (0.09) c	0.07 (0.06) b
		Non-Bt isoline	0.22 (0.09) ab	39.33 (0.10) b	38.53 (0.13) a
	No-spray	BARI Bt begun-1	0.00 (0.00) b	0.00 (0.00) c	0.00 (0.00) b
		Non-Bt isoline	0.65 (0.21) a	50.85 (0.10) a	49.29 (0.20) a
2017	Spray	BARI Bt begun-1	0.02 (0.07) b	0.00 (0.00) c	0.00 (0.00) b
		Non-Bt isoline	12.94 (0.08) a	41.52 (0.12) b	27.78 (0.13) a
	No-spray	BARI Bt begun-1	0.00 (0.00) b	0.00 (0.00) c	0.00 (0.00) b
		Non-Bt isoline	15.56 (0.13) a	52.43 (0.08) a	35.25 (0.15) a

^1^ Figures in parenthesis are SE values; means followed by the same letter in a column within a year do not significantly differ from each other at 5% level by HSD

#### Yields and gross margins

In 2016 Bt begun-1 had a higher gross margin than its isoline. Spraying Bt begun-1 improved the gross margin to $2,962.94 /ha compared to not spraying Bt begun-1 ($939.42/ha), despite the increased cost of production (Table 7). Likewise, in 2017 spraying Bt begun-1 improved the gross margin from $1,289.78 to $3,654.59. In both years, not spraying the non-Bt line resulted in a negative gross margin.

**Table 7:** Mean yields and economic returns^1^ in brinjal by brinjal shoot and fruit borer (BFSB) relative to insecticides and varieties, OFRD, BARI, Bogra, Bangladesh.

Year	Spray schedule	Variety	Fruit yield (t/ha)		Gross return ($)/ha^2^	Gross margin ($)/ha^2^
			Non-infested fruit	Infested fruit		
2016	Spray	BARI *Bt* begun-1	17.55 (0.64) a	0.06 (0.05) c	5,221.12 (189.92) a	2,962.94 (189.92) a
		Non-*Bt*isoline	9.29 (0.29) b	4.55 (0.53) a	2,872.45 (86.31) b	614.27 (86.31) b
	No-spray	BARI *Bt* begun-1	6.99 (0.72) c	0.0006 (0.0006) c	2,078.62 (213.78) c	939.42 (213.78) b
		Non-*Bt*isoline	2.59 (0.29) d	1.59 (0.08) b	807.23 (88.69) d	-331.97 (88.69) c
2017	Spray	BARI *Bt* begun-1	19.88 (1.25) a	0.00 (0.00) c	5,912.77 (371.76) a	3,654.59 (371.76) a
		Non-*Bt* isoline	8.73 (0.80) b	8.48 (0.71) a	2,799.82 (252.03) b	541.64 (252.03) bc
	No-spray	BARI*Bt* begun-1	8.16 (0.66) b	0.00 (0.00) c	2,428.99 (195.26) b	1,289.78 (195.26) b
		Non-*Bt* isoline	2.63 (0.32) c	2.55 (0.16) b	844.61 (99.34) c	-294.59 (99.34) c

^1^ Figures in parenthesis are SE values; means followed by the same letter in a column within a year do not significantly differ from each other at 5% level by HSD

^2^ Market price of brinjal: Non-infested BARI Bt begun-4 @ $0.36/kg, others @ $0.30/kg and infested @ $0.02/kg. Cost of spray: two laborers/spray/ha @ Tk. $3.56/labour; Urea@ $0.19/kg, TSP@ $0.26/kg, MP@ $0.18/kg, Zypsum @ $0.07/kg, Boric acid @ $1.78/kg, Zn @ @3.96/kg, Cowdung @ $0.01/kg. Admire @ $17.8/250 ml and Proclaim @ 26.77/500 ml. 1 US dollar = 84.05 Bangladeshi Taka (29 March 2018)

#### Effects on non-target pest arthropods

Eleven non-target pest arthropods species were observed on leaves in 2016, with whiteflies, aphids, thrips, jassids, flea beetles and mites having sufficient populations for meaningful analysis (Table 8). Only with jassids and mites did variety have a significant effect, but it was not consistent. Spraying increased whiteflies abundance in both years, but this was not the case with most other species.

**Table 8:** Mean abundance^1^ of non-target pest arthropods (No./leaf) in brinjal relative to insecticides and varieties, OFRD, BARI, Bogra, Bangladesh.

Year	Spray schedule	Variety	Whitefly^2^	Aphid^3^	Thrips^4^	Jassid^5^	Flea beetle^6^	Mite^7^
2016	Spray	BARI *Bt* begun-1	2.28 (0.15) a	0.81 (0.07) b	1.13 (0.03) b	0.23 (0.02) c	0.020 (0.005) a	5.65 (0.41) b
		Non-*Bt*isoline	2.72 (0.39) a	0.80 (0.07) b	1.12 (0.08) b	0.24 (0.03) c	0.022 (0.005) a	6.90 (0.57) a
	No-spray	BARI *Bt* begun-1	0.85 (0.06) b	2.12 (0.19) a	2.32 (0.11) a	0.85 (0.05) b	0.028 (0.012) a	2.22 (0.42) c
		Non-*Bt*isoline	0.82 (0.03) b	2.18 (0.13) a	2.43 (0.06) a	0.97 (0.04) a	0.025 (0.011) a	2.56 (0.12) c
2017	Spray	BARI *Bt* begun-1	0.849 (0.06) a	0.152 (0.01) b	1.38 (0.12) a	0.263 (0.02) b		
		Non-*Bt* isoline	1.047 (0.07) a	0.119 (0.03) b	1.33 (0.07) a	0.335 (0.01) b		
	No-spray	BARI *Bt* begun-1	0.449 (0.01) b	0.361 (0.02) a	1.19 (0.08) a	0.695 (0.01) a		
		Non-*Bt* isoline	0.490 (0.01) b	0.367 (0.04) a	1.22 (0.12) a	0.708 (0.03) a		

^1^ Figures in parenthesis are SE values; means followed by the same letter in a column within a year do not differ significantly by HSD at 5% level

^2^*Bemisia tabaci* Gennadius,

^3^*Aphis gossypii* Glover,

^4^*Thrips palmi* Karny,

^5^*Amrasca biguttula biguttula* Ishida,

^6^*Phyllotreta striolata*,

^7^*Tetranychus urticae* Koch

Populations of mirid bug (*Helopeltis* sp.), monolepta beetles (*Monolepta* sp), shield bug (*Scutiphora* sp.), leaf miner (*Liriomyza*sp.) and green leafhopper (*Nephotettix* sp.) were too low (< 0.01) for meaningful analysis. In 2017, populations of flea beetle (*Phyllotreta striolata*), Mirid bug (*Helopeltis* sp.), Epilachna (*Epilachna* sp.), Bombardier beetle (*Pheropsophus* sp.), Hooded hopper (*Oxyrachis terandus* Fab.), Semiloper (*Trichoplusia* sp.), mites (*Tetranychus urticae* Koch) and green leaf hopper (*Nephotettix* sp.) were too low (< 0.01) for meaningful analysis.

In 2016, there were only six non-target species of pest arthropods caught in pitfall traps: flea beetles, grasshoppers, monolepta beetles, termites, June beetles and stink bugs, and their populations were all too low for meaningful analysis. Sticky traps captured aphids, whiteflies, flea beetles and jassids but populations were too low for meaningful analysis. Similar results were observed in 2017 with all different sampling methods.

#### Effects on non-target beneficial arthropods

As in the first experiment, lady beetles and spiders were the most abundant beneficial arthropods in 2016 on plant samples, but only reached a peak of 0.04 beetles/leaf and 0.029 spiders/leaf (Table 9). Spraying did not have a consistent effect on reducing the densities of either beneficial in either year. Populations of red ant (*Solenopsis* sp.), small black ant (*Camponotus* sp.), rove beetle (*Homaeotarsus* sp.), ground beetle (*Ophionia nigrofasciata* sp.) and syrphid fly (*Syrphus* sp.), honeybee (*Apis* sp.) and bombardier beetle (*Pheropsophus* sp.) were too low (<0.01) for meaningful analysis. For pitfall traps, the numbers of beneficial arthropods captured were too low (<0.1/trap) for meaningful analysis.

**Table 9:** Mean abundance^1^ of non-target beneficial arthropods (No./leaf) in brinjal relative to insecticides and varieties, OFRD, BARI, Bogra, Bangladesh.

Year	Spray schedule	Variety	Ladybird beetle^2^	Spider^3^
2016	Spray	BARI *Bt* begun-1	0.01 (0.001) c	0.017 (0.004) ab
		Non-*Bt*isoline	0.02 (0.003) bc	0.014 (0.002) b
	No-spray	BARI *Bt* begun-1	0.04 (0.004) a	0.029 (0.003) a
		Non-*Bt*isoline	0.03 (0.007) ab	0.023 (0.001) ab
2017	Spray	BARI *Bt* begun-1	0.006 (0.002) a	0.038 (0.001) a
		Non-*Bt* isoline	0.005 (0.002) a	0.036 (0.004) a
	No-spray	BARI *Bt* begun-1	0.013 (0.002) a	0.044 (0.005) a
		Non-*Bt* isoline	0.012 (0.002) a	0.051 (0.006) a

^1^ Figures in parenthesis are SE values; means followed by the same letter in a column within a year do not differ significantly by HSD at 5% level

^2^*Coccinella* sp.,

^3^*Oxyopes* sp. plus *Tetragnatha* sp.

Yellow sticky traps caught flying insects in the crop canopy but the numbers were low, with the highest counts being ladybird beetles at 0.446 per trap/wk. (Table 10). In neither year were populations affected by insecticide sprays or brinjal variety. Populations of rove beetle (*Homaeotarsus* sp.), damsel fly (*Agriocnemis* sp.) and ground beetle (*Ophionia nigrofasciata*) were too low (<0.01) for meaningful analysis.

**Table 10:** Mean abundance^1^ of non-target beneficial arthropods (No./trap) recovered from yellow sticky traps in brinjal relative to insecticides and varieties, OFRD, BARI, Bogra, Bangladesh.

Year	Spray schedule	Variety	Ladybird beetle^2^	Spider^3^	Small black ant^4^	Carabid beetle^5^
2016	Spray	BARI *Bt* begun-1	0.037 (0.005) a	0.0006 (0.0003) a	0.003 (0.002) a	NA
		Non-*Bt*isoline	0.038 (0.007) a	0.0006 (0.0003) a	0.006 (0.001) a	NA
	No-spray	BARI *Bt* begun-1	0.039 (0.004) a	0.0014 (0.001) a	0.007 (0.002) a	NA
		Non-*Bt*isoline	0.039 (0.006) a	0.0006 (0.0003) a	0.006 (0.003) a	NA
2017	Spray	BARI *Bt* begun-1	0.309 (0.068) a	0.000 (0.00) a	0.108 (0.069) a	0.064 (0.03) a
		Non-*Bt* isoline	0.304 (0.049) a	0.025 (0.024) a	0.083 (0.027) a	0.113 (0.17) a
	No-spray	BARI *Bt* begun-1	0.446 (0.063) a	0.005 (0.005) a	0.127 (0.028) a	0.127 (0.07) a
		Non-*Bt* isoline	0.446 (0.057) a	0.000 (0.00) a	0.167 (0.041) a	0.073 (0.09) a

^1^ Figures in parenthesis are SE values; means followed by the same letter in a column within a year do not differ significantly by HSD at 5% level HSD

^2^*Coccinella* sp.,

^3^*Oxyopes* sp. plus *Tetragnatha* sp.

^4^*Camponotus* sp.,

^5^*Pterostichus* sp.; NA, data not taken because of extremely low populations.

#### Environmental impact quotient (EIQ)

The same number of sprays (19) was applied to sprayed plots in both years. The seasonal insecticide load/ha for imidacloprid and emamectin benzoate was 36.7 and 26.3 mg active ingredient per ha, respectively, and the seasonal calculated EIQ values for imidacloprid were 8.4 (consumer), 5.6 (field worker) and 75.5 (ecological) and for emamectin benzoate 1.7 (consumer), 3.8 (farmer worker) and 27.9 (ecological). This was the same as in the first experiment with four varieties.

## Discussion

These studies present the first replicated field trials assessing the four Bt brinjal varieties that were first introduced to Bangladesh farmers in 2014. Collectively, the results demonstrate that the four Bt varieties provided more fruit and nearly complete protection from infestation by BFSB, compared to their non-Bt isolines, even when no insecticide sprays were applied. Most importantly, these studies revealed that all Bt lines had higher gross returns than their non-Bt isolines.

The insecticide spray regime of using imidacloprid and emamectin benzoate on non-Bt brinjal was unable to decrease the level of BFSB infestation to the level achieved by using its Bt brinjal isoline. Furthermore, it is worth noting that spraying these insecticides tended to increase the infestation in non-Bt fruit. For example, in Table 1 for 2016 the average percent of infested fruit by weight of all four non-Bt lines was 28.2% when they were sprayed and only 16.5% when not sprayed. In 2017 the same phenomenon occurred with an average infestation of 38.4% when sprayed and only 18.9% when not sprayed. One hypothesis for this phenomenon is that spraying reduced the natural enemy population of BFSB and thus increased damage to the brinjal. Further work is needed to confirm this hypothesis.

When comparing the arthropod communities in Bt and non-Bt brinjal, we were not able to detect any differences in numbers of either non-target pest species or beneficial species, suggesting that the four Bt brinjal varieties control the most important insect pest of brinjal in Bangladesh, BFSB, without disrupting arthropod biodiversity. However insecticide sprays did have a disruptive effect on some species of beneficial arthropods and this could support the hypothesis proposed above.

It is important to note that the yield of Bt brinjal, based on weight of the fruit, was improved with the insecticide spray regime. It appears that arthropods such as whiteflies, mites, jassids and aphids, none of which are susceptible to Cry1Ac, still need to be managed. Scheduled applications of the two insecticides, without regard to any threshold, resulted in a relatively high EIQ. The next challenge will be to develop thresholds for the common sucking arthropods encountered in Bangladesh using selective insecticides that will not disrupt biological control agents of BFSB. These experiments are currently underway.

These results from Bangladesh are similar to those from studies conducted in the Philippines in which event EE-1, the same event used to create the four Bt brinjal varieties used in these studies, was incorporated into open pollinated lines and provided almost complete control of BFSB in different locations over three cropping periods [10]. Furthermore, additional ecological studies in the Philippines [11] documented that many arthropod taxa are associated with Bt eggplants and their non-Bt comparators, but found few significant differences in seasonal mean densities of arthropod taxa between Bt and non-Bt eggplants when no insecticides were used. Principal Response Curve analyses showed no statistically significant impact of Bt eggplants on overall arthropod communities through time in any season. Furthermore, the Philippine studies found no significant adverse impacts of Bt eggplants on species abundance, diversity and community dynamics, particularly for beneficial NTOs. Similarly, in the present study we did not find any differences in the arthropod communities in any Bt brinjal variety compared to its non-Bt isoline. This is not surprising because the ecological effect of Cry1Ac has been extensively studied and shown to have little to no effect on non-target organisms outside of the Lepidoptera [12–24].

In most cases, statistically higher numbers of non-target pest arthropods were observed in no-spray plots irrespective of varieties, except for whitefly and mites. Furthermore, populations were similar in Bt and non-Bt isolines, irrespective of spray regime in most cases. Similar patterns have been observed before. In India, it has been reported that populations of major non-target insect pests (leafhoppers, whiteflies, ash weevils, aphids, dusky and red cotton bug, and green bug) and generalist predators (ladybirds, chrysopids, and spiders) did not differ significantly between Bt and non-Bt cotton lines, while their numbers were lower in insecticide protected than under unprotected conditions, except for aphids and whiteflies [25]. Ladybird beetles were more abundant in no-spray plots but similar abundances of non-target beneficial and other arthropods were observed in Bt and non-Bt isolines irrespective of spray regime in most cases. Other larger scale studies and meta-analyses have documented that Bt crops were much safer to non-target organisms than the alternative use of traditional insecticides for control of the pests targeted by the Bt proteins [17, 19, 21, 26].

Plot sizes in this study were relatively small and the effect of this on study outcomes would depend somewhat on the general mobility of the species examined. While specific guidance on plot size for any given study is not available, there is general agreement that plots should be as large as possible to avoid inter-plot exchanges of arthropods [27, 28]. In reality, plot size is typically dictated by experimental design issues, and space resources as they were here. Nonetheless, despite small plots sizes, this study clearly delineated the effects of Bt eggplant on target pest abundance and its associated impact on yield. Furthermore, other experiments and research syntheses that have examined a wide range of plots sizes have shown that plot size has a relatively small impact on the assessment of non-target effects [17, 29]. Finally, the very small scale of individual farms growing eggplant in Bangladesh suggests that our studies are reflective of commercial practices, and thus of potential non-target effects.

Since their first introduction in 1996, biotech crops have been large scale field crops except for the relatively small-scale production of virus resistant papaya and squash and insect-resistant sweet corn, and the brief commercialization of Bt potatoes [3]. Bt brinjal is the first Bt vegetable crop introduced into a developing country and the results reported here indicate that it can be highly successful. An ex-ante study in 2005 estimated that the introduction of Bt eggplant into Bangladesh would result in a decrease of insecticides by 70–90%, increase yield by 15–30% and increase the gross return by 37–64% [30]. The data from this study supports these general predictions.

To realize these benefits of Bt brinjal for the long term, it is critical that EFSB does not rapidly evolve resistance to the Cry1Ac protein it produces. A government condition for the release of Bt brinjal in Bangladesh requires planting a refuge of non-Bt brinjal and training materials provided to farmers emphasizing that non-Bt brinjal should be planted around Bt fields as a structured refuge. In addition, to this refuge, there are many non-Bt varieties commonly grown in Bangladesh that also may serve as a natural refuge for resistance management. Nonetheless, it will be important to develop lines that express multiple Bt proteins because this is another key factor in delaying the evolution of resistance [31].

In conclusion, the four varieties of Bt eggplant examined here provide high levels of BFSB control, demonstrate higher gross returns than their non-Bt isolines and have the potential to greatly reduce insecticide inputs and their associated costs for management of this devastating pest. Additional controls of other pests in the system appear to be important and further development of management strategies for these will likely lead to further favorable economic returns in crop production for farmers growing brinjal in Bangladesh. In addition to providing excellent pest suppression, cultivation of Bt brinjal demonstrated no undesirable non-target effects on other arthropods in the system, especially those beneficial organisms that contribute important ecosystem services like biological control. Overall, careful stewardship will be critical to preserving this valuable pest control technology as adoption continues to increase from the more than 27,000 farmers who grew Bt brinjal in 2018 [32].
